# Laser ablation under intraductal cholangioscopic guidance for cholangiocarcinoma

**DOI:** 10.1055/a-2051-7984

**Published:** 2023-03-30

**Authors:** Mingxing Xia, Xianrong Hu, Ting Zhang, Jinbing Sun, Cui Chen, Bing Hu

**Affiliations:** Department of Gastroenterology and Endoscopy, The Third Affiliated Hospital of Naval Medical University, Shanghai, China

An 82-year-old man was admitted for progressively worsening jaundice. Magnetic resonance cholangiopancreatography (MRCP) showed a stenosis of 2 cm in length in the common bile duct, with dilatation of the biliary system above ( Fig. 1 ). Endoscopic retrograde cholangiopancreatography (ERCP)-guided cytologic brushing confirmed adenocarcinoma. The patient refused surgery owing to his age and significant co-morbidities. Another ERCP with intraductal cholangioscopic (SpyGlass) observation was arranged. Some mucosal erosions and nodular hyperplasia were found endoscopically ( Fig. 2 ). A circumferential irradiation fiber was introduced through the channel of the endoscope ( Fig. 3 ) and connected to a laser system (Leonardo, 1470 nm and 980 nm dual wavelength; CeramOpteec GmbH of Biolitec AG, Bonn, Germany). Continuous ablation was carried out until all visualized tumors had been burned to a whitish and necrotic appearance, producing a wider lumen ( Fig. 4 ), before a metal stent was placed ( Fig. 5 ). The patient recovered unremarkably and was discharged 5 days later ( Video 1 ).

**Fig. 1:**
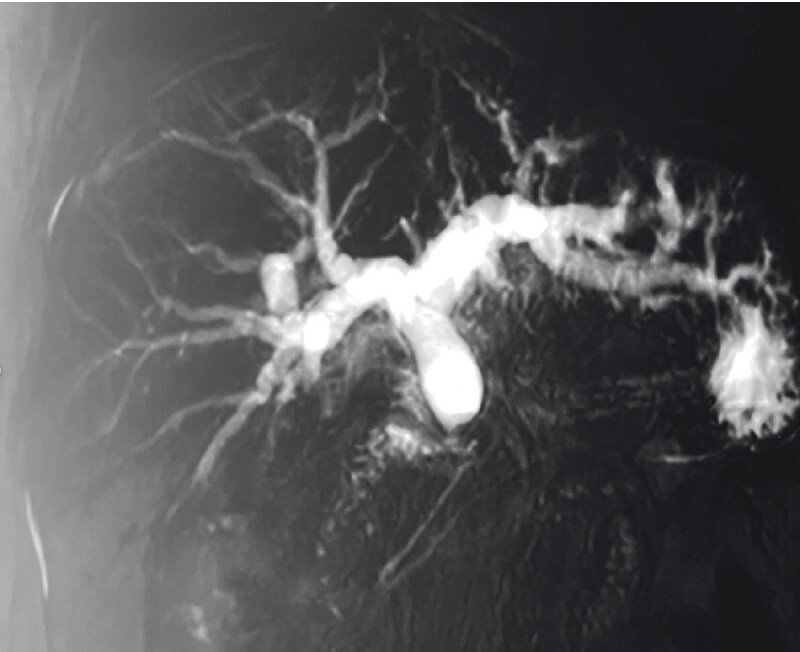
Magnetic resonance cholangiopancreatography image showing a stenosis of 2 cm in length in the common bile duct, with dilatation of the biliary system above.

**Fig. 2:**
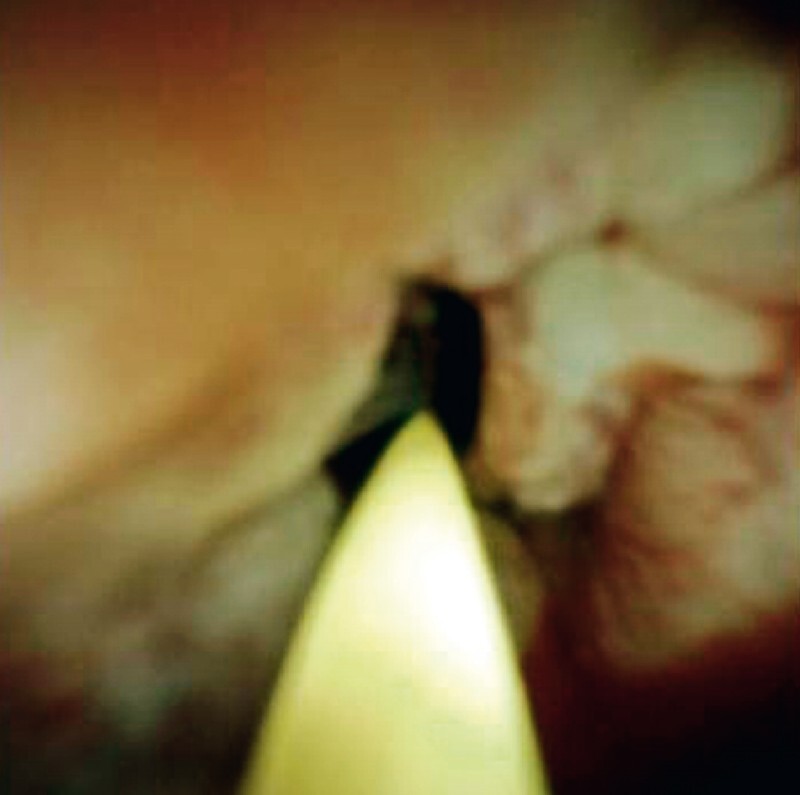
Intraductal cholangioscopic view showing some mucosal erosions and nodular hyperplasia in the common bile duct.

**Fig. 3:**
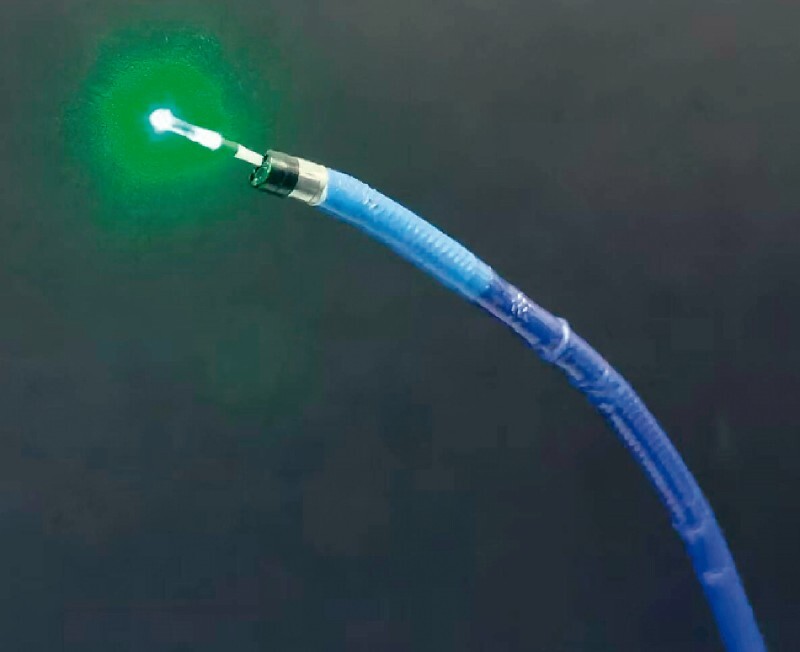
Photograph of the laser fiber after its introduction through the channel of the intraductal cholangioscope.

**Fig. 4:**
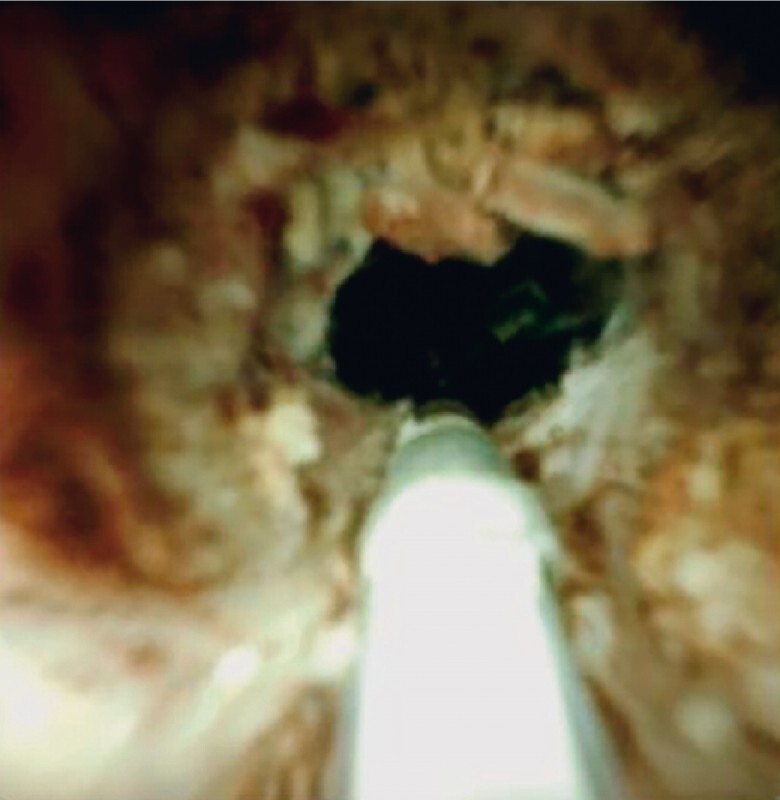
Intraductal cholangioscopic view showing the tumor tissues burned whitish and necrotic by laser ablation, with the wider lumen now apparent.

**Fig. 5:**
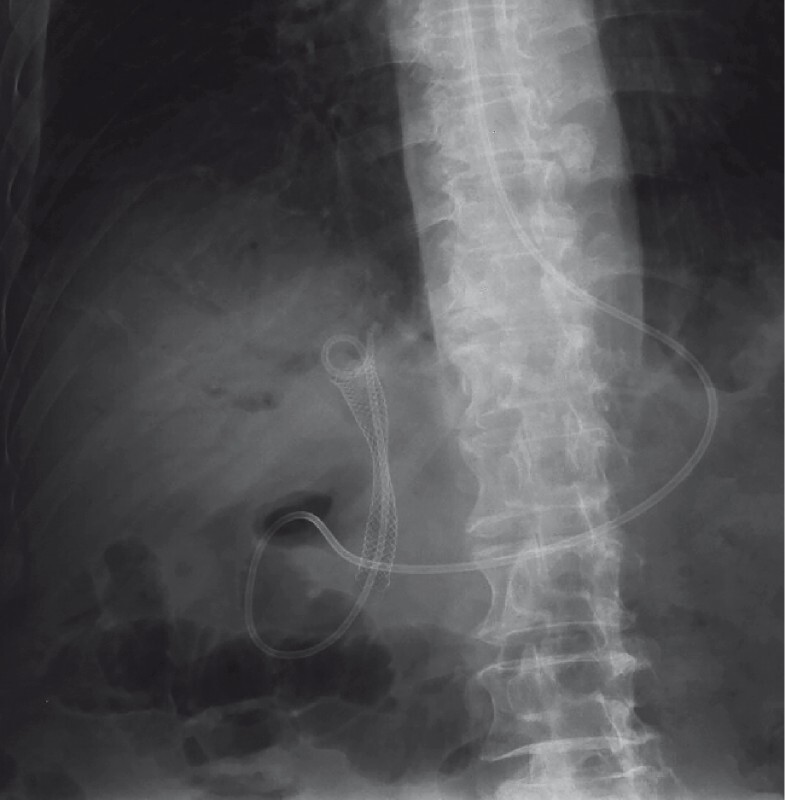
Fluoroscopic view showing a metal stent that had been endoscopically placed across the biliary stricture.

**Video 1** Laser ablation under intraductal cholangioscopic guidance is performed for a cholangiocarcinoma.


In recent decades, endoscopic radiofrequency ablation has been introduced into clinical use to palliate unresectable cholangiocarcinoma 1 2 ; however, it has not been possible to perform this technique under direct visualization. A preclinical study has been reported on laser ablation in the biliary system 3 . A few authors have also reported using intraductal cholangioscopy-guided laser dissection for benign pancreatic/biliary ductal strictures 4 5 . To the best of our knowledge, this may be the first application of visualized ablation for cholangiocarcinoma using a dual-wavelength laser such as this. Our experience suggested good cutting and hemostatic effect for this novel technique, which may be promising in future applications.

Endoscopy_UCTN_Code_TTT_1AR_2AF

## Notes

Endoscopy E-Videos

https://eref.thieme.de/e-videos
*Endoscopy E-Videos* is an open access online section, reporting on interesting cases and new techniques in gastroenterological endoscopy. All papers include a high quality video and all contributions are freely accessible online. Processing charges apply, discounts and wavers acc. to HINARI are available. This section has its own submission website at https://mc.manuscriptcentral.com/e-videos

## References

[1] Yang J Wang J Zhou H Efficacy and safety of endoscopic radiofrequency ablation for unresectable extrahepatic cholangiocarcinoma: a randomized trial Endoscopy 2018 50 751 760 29342492 10.1055/s-0043-124870

[2] Gao D J Yang J F Ma S R Endoscopic radiofrequency ablation plus plastic stent placement versus stent placement alone for unresectable extrahepatic biliary cancer: A multicenter randomized controlled trial Gastrointest Endosc 2021 94 91 10000 33359435 10.1016/j.gie.2020.12.016

[3] Saccomandi P Quero G Gassino R Laser ablation of the biliary tree: in vivo proof of concept as potential treatment of unresectable cholangiocarcinoma Int J Hyperthermia 2018 34 1372 1380 29322853 10.1080/02656736.2018.1427287

[4] Mittal C Shah R J Pancreatoscopy-guided laser dissection and ablation for treatment of benign and neoplastic pancreatic disorders: an initial report (with videos) Gastrointest Endosc 2019 89 384 389 30176224 10.1016/j.gie.2018.08.045

[5] Han S Shah R J Cholangiopancreatoscopy-guided laser dissection and ablation for pancreas and biliary strictures and neoplasia Endosc Int Open 2020 8 E1091 E1096 32743063 10.1055/a-1192-4082 PMC7373658

